# Testicular microlithiasis as a familial risk factor for testicular germ cell tumour

**DOI:** 10.1038/sj.bjc.6604060

**Published:** 2007-10-30

**Authors:** J Coffey, R A Huddart, F Elliott, S A Sohaib, E Parker, D Dudakia, J L Pugh, D F Easton, D T Bishop, M R Stratton, E A Rapley

**Affiliations:** 1 Testicular Cancer Genetics Team, Section of Cancer Genetics, Institute of Cancer Research, Sutton, Surrey, UK; 2 Academic Radiotherapy Unit, Institute of Cancer Research, Sutton, Surrey, UK; 3 Section of Epidemiology & Biostatistics, Leeds Institute of Molecular Medicine, St James's University Hospital, Leeds, UK; 4 Department of Diagnostic Radiology, The Royal Marsden Hospital, Sutton, Surrey, UK; 5 Cancer Research UK, Genetic Epidemiology Unit, Strangeways Research Laboratory, Cambridge, UK

**Keywords:** testicular germ cell tumour, testicular microlithiasis, genetic susceptibility

## Abstract

Testicular microlithiasis (TM) is characterised by small intratesticular calcifications, which can be visualised by ultrasound. Men with testicular germ cell tumour (TGCT) have a higher frequency of TM than men without TGCT. To clarify the association between TGCT and TM and to investigate the relationship between TGCT susceptibility and TM, we recruited TGCT patients with and without family history of TGCT, unaffected male relatives and healthy male controls from the UK. Testicular ultrasound data were analysed from 328 men. Testicular microlithiasis was more frequent in TGCT cases than controls (36.7 *vs* 17.8%, age adjusted *P*<0.0001) and in unaffected male relatives than controls (34.5 *vs* 17.8%, age adjusted *P*=0.02). Testicular germ cell tumour case and matched relative pairs showed greater concordance for TM than would be expected by chance (*P*=0.05). We show that TM is present at a higher frequency in relatives of TGCT cases than expected by chance indicating that TM is a familial risk factor for TGCT. Although the familiality of TM could be due to shared exposures, it is likely that there exists a genetic susceptibility to TM that also predisposes to TGCT. We suggest that TM is an alternative manifestation of a TGCT susceptibility allele.

Testicular germ cell tumour (TGCT) is the most common cancer in men aged 15–45 years. Risk factors for TGCT include a family history of disease (Forman *et al*, 1992; Westergaard *et al*, 1996; Heimdal *et al*, 1996a; Sonneveld *et al*, 1999; Hemminki and Li, 2004), a previously diagnosed germ cell tumour (Osterlind *et al*, 1991; Wanderas *et al*, 1997), a history of undescended testis (Brown *et al*, 1987; Swerdlow *et al*, 1997), infertility (Petersen *et al*, 1998; Moller and Skakkebaek, 1999; Jacobsen *et al*, 2001; Richiardi *et al*, 2004), atrophy (Harland *et al*, 1998), and gonadal dysgenesis (Verp and Simpson, 1987). In a proportion of cases (∼2%), a first-degree family member is also affected with the disease (Forman *et al*, 1992). The relative risk to a brother of a TGCT case is 8–10 (Forman *et al*, 1992; Heimdal *et al*, 1996b; Hemminki and Li, 2004), which is higher than for most other cancer types that rarely exceed four (Dong and Hemminki, 2001) and suggests that predisposition genes are important in this disease. However, despite intensive efforts, susceptibility genes are yet to be identified (Crockford *et al*, 2006).

Testicular microlithiasis (TM), the presence of multiple small deposits of calcium within the testis, shows characteristic sonographic findings of multiple, intratesticular, nonshadowing echogenic foci. Since the original description of ultrasound-detected TM (Doherty *et al*, 1987), a number of studies have reported an association between TGCT and TM (Backus *et al*, 1994; Miller *et al*, 1996, 2006; Cast *et al*, 2000; Bach *et al*, 2001; Bennett *et al*, 2001; Derogee *et al*, 2001; Middleton *et al*, 2002; Sakamoto *et al*, 2006). All the studies report ultrasound findings for men investigated for a suspect testicular pathology (infertility, hydrocele, varicocele or suspected tumour) and compare the rate of TM in men found to have TGCT with those that have other diagnoses, including normal testes. The studies have shown a high frequency of TM associated with TGCT (range 12–74%) compared to 0.68–9% for other diagnoses (Rashid *et al*, 2004). No study has compared TGCT cases to control subjects without suspected testicular pathology. Two studies that examined the prevalence of TM among non-symptomatic army recruits (median age at ultrasound of 18–20 years) showed approximately 6% had TM (defined as ⩾5 microliths) (Peterson *et al*, 2001; Serter *et al*, 2006).

Although previous data suggest that TM is a risk factor for TGCT, the frequency of TM in relatives of TGCT patients has never been examined. Furthermore, the familiality of TM itself has not previously been investigated. Given that the relationship of TM to the well-documented familial risk of TGCT is unknown, we investigated the frequency of TM among TGCT cases, their male relatives and healthy male controls to evaluate the hypothesis that TM is a familial risk factor for TGCT and to examine evidence of familial aggregation of TM.

## MATERIALS AND METHODS

### Participants

Cases of TGCT were identified from the Royal Marsden Hospital testicular cancer clinic, Sutton, Surrey, UK and from a national UK study of familial testicular cancer coordinated at the Institute of Cancer Research (ICR). All TGCT cases were unrelated. In cases with a family history of TGCT, the affected relative(s) were not recruited. All cases had a prior diagnosis of germ cell tumour and were diagnosed between 1973 and 2005. Tumour histology was confirmed from pathology reports or medical notes. Each case was asked to nominate an unaffected relative (preferably first degree) to participate in the study and a friend or in-law to act as a control subject. To prevent intrafamilial correlation, only one unaffected relative per TGCT case was recruited. Additional controls were identified by approaching local sporting clubs and by a call for volunteers at the ICR. Only patients of white Caucasian ancestry were included in this analysis. All men participated with fully informed consent and local ethical review board approval.

### Procedures

Study participants attended a research clinic prior to which they were asked to complete a family history questionnaire and a medical history questionnaire. These documents were reviewed at the research clinic. In addition, height, weight, BMI, percentage body fat and blood pressure were recorded.

Testicular ultrasound scans were carried out on both testes in men without previous orchidectomy, on the solitary remaining testis in those with a previous unilateral orchidectomy and on residual testicular tissue in those men with bilateral tumours who had undergone initial orchidectomy and subsequent contralateral partial orchidectomy. All scans were carried out by a single sonographer (EP) using 15–17.5 MHz linear array probes (Philips iU 22 system; Koninklijke Philips Electronics NV and ATL HDI system, Bothell, WA, USA). Images were stored on a hard drive and all clinically significant findings were checked by a consultant radiologist (AS) who also double-read scans at the beginning of the study as a quality assurance measure. Ultrasound was performed in a semiblinded manner. The sounographer was aware if the research participant had a previous germ cell tumour and orchidectomy but was unaware if the TGCT case had a family history of disease or not. For all other research participants, the sounographer was not aware if the participant was an unaffected male relative of a TGCT case or a healthy male control. For each examinable testis, TM was counted, categorised and recorded as shown in Table 1, based on the model described by Backus *et al*, (1994). We performed analyses based on any TM (⩾1 microlith in a testes) also described as Limited TM (LTM) and on TM ⩾5 microliths per testes, referred to as Classical TM (CTM) (Middleton *et al*, 2002).

### Statistical analysis

Participants were categorised into three groups; TGCT case (cases), unaffected male relative of a TGCT case (relatives) and healthy male volunteers with no known family history of TGCT (controls). For more detailed analysis, the first two groups were subclassified as follows: TGCT-FH (family history), an index case with a germ cell tumour and at least one other affected male relative with the disease; TGCT-S (sporadic), an index case with a germ cell tumour and no known family history of the disease; relative-FH, an unaffected male relative of a TGCT-FH case (i.e. a participant with at least two affected male relatives); relative-S, an unaffected male relative of a TGCT-S case (i.e. with only one affected male relative) (Table 2).

Mean age at ultrasound was calculated for each of the three groups and compared by *t*-test. Differences in TM frequency among study groups were compared using unconditional logistic regression, adjusting for age as a continuous covariate. Concordance for TM among matched relative pairs was tested using the Pearson *χ*^2^ test. All statistical analyses were carried out using STATA (StataCorp. 2005. Stata Statistical Software: Release 9. College Station, TX: StataCorp LP).

## RESULTS

Between June 2004 and June 2006, 328 men consented, were examined by ultrasound and included in the data analysis. Of these, 169 were cases, 58 were relatives, and 101 were controls (Table 2). Forty-eight TGCT cases had a relative who participated in the study (26 (54.2%) brothers, 7 (14.5%) fathers, 13 (27.1%) sons, 1 (2.1%) maternal uncle and 1 (2.1%) nephew). For 10 relatives, there was no matching case available for analysis. The reasons were: in four relatives, the case had bilateral disease and therefore did not have testes available for ultrasound; in two relatives, the case was deceased; and in four relatives, the case consented to the study but failed to attend the research clinic.

A total of 493 testes in 328 men were examined by ultrasound. Of 169 cases, 162 had only one testis available for ultrasound examination. Four cases had bilateral disease and had undergone a standard total orchidectomy for the first tumour. In two of these cases, the second tumour was diagnosed as CIS at biopsy and both patients were treated without contralateral orchidectomies; ultrasound was performed on the remaining testes. The other two patients with bilateral disease had a contralateral partial orchidectomy and the remaining testicular tissue was examined by ultrasound. Seven cases did not have orchidectomies (five with extragonadal germ cell tumours without clear gonadal primaries and two patients treated for metastatic disease without orchidectomy), both testes were examined by ultrasound in these seven cases. Of 58 relatives, one participant had a history of testicular torsion and orchidectomy; all other relatives had both testes available for analysis. No orchidectomies were reported in the 101 controls (Table 2). The mean age of the controls was significantly lower (42) than that of the cases (47) (*P*=0.0003, *t*-test) and approached significance with the unaffected relatives (45) (*P*=0.14, *t*-test); there was no age difference between the cases and their relatives (*P*=0.3, *t*-test).

A total of 173 tumours were reported in the 169 cases as four patients had a second tumour (two seminomas and two CIS). Tumour histology for the first tumour is given in Table 3. A total of 41 (24.0%) cases had a documented family history of TGCT. The mean age of diagnosis was 34 years for cases both with and without a family history (Table 3). The median time between TGCT diagnosis and research ultrasound was 12 years (range 1–32).

Testicular microlithiasis (⩾1 microlith in any testis) was more frequent in cases than controls, 62/169 (36.7%) *vs* 18/101 (17.8%), odds ratio (OR) 3.0 (95% CI 1.6–5.6), age adjusted *P*<0.0001. The frequency in unaffected male relatives (20/58, 34.5%) was also higher than that in controls (OR 2.5, 95% CI 1.2–5.4, age adjusted *P*=0.02). The proportion of cases and relatives with TM was not significantly different. However, the number of testes available for study in the relative group is double that of the cases. Comparing the testis examined in cases with the same testis in the matched relative, 48 cases had assessable testis of which 18/48 (37.5%) had TM and 11/48 (22.9%) relatives matched testes had TM (*P*⩽0.2).

For the 48 families with a case and a relative, 10 pairs were concordant for the presence of TM, 22 were concordant for the absence of TM and 16 were discordant (*P*=0.05 for independence) showing that TM diagnosis aggregates in families. To determine if the familial aggregation was likely to be due to shared environmental or shared genetic factors, we evaluated the proportion of brother pairs and other relative pairs in the concordant/discordant TM groups. Of the TM concordant series, 6 were brother pairs (60%); in the discordant set, 11 (69%) were brothers and in the no TM concordant set, 9 (41%) brothers (*P*=0.22 for equal distribution between brother and other relative pairs).

The frequency of TM in TGCT cases with a family history of TGCT compared with cases with no family history was 18/41 (43.9%) *vs* 44/128 (34.4%); age adjusted *P*=0.3. The frequency of TM in men with two or more relatives with TGCT than men with only one affected relative was 11/24 (45.8%) *vs* 9/34 (26.5%); age adjusted *P*=0.1 (Table 4).

The frequency of bilateral TM was not different between relatives and controls with TM (8/20 (40.0%) *vs* 8/18 (44.4%), *P*=0.8). The severity of TM was greater in cases and relatives than for controls. A total of 83/101 (82%) of controls had no TM, 12 (12%) had LTM and 6 (6%) had CTM. In comparison, 145/227 (64%) of cases and relatives combined had no TM, 39 (17%) had LTM and 43 (19%) had CTM (*χ*^2^=12.54, *P* trend=0.0004).

There was no difference in the distribution of histological types between TGCT cases with and without TM. In cases with TM, 46.7% were diagnosed with a seminoma and 53.2% with non-seminoma; in cases without TM, 44.3% were diagnosed with seminoma and 55.6% with non-seminoma.

Of the four patients with bilateral TGCT and an assessable testis, both patients with biopsy-proven CIS in the contralateral testis showed TM (one LTM and one scant TM). In the two patients who had undergone partial orchidectomy, one had LTM in the remaining portion of the testis and the second had no TM. Of the seven TGCT cases with two assessable testes, bilateral TM was documented in one case, who had a metastatic seminoma treated without orchidectomy (Table 3). In the five patients with extragonadal GCT treated without orchidectomies, four had unilateral LTM.

Two tumours were diagnosed in the course of the study. A brother of a TGCT-S case showed bilateral LTM and a lesion that was suspected to be a tumour on ultrasound. Orchidectomy revealed a classical seminoma. The second tumour was diagnosed in an index TGCT-S case. This patient was treated for metastatic disease without orchidectomy 15 years prior to the study and showed no TM on ultrasound but a calcified lesion in the left testis. Orchidectomy revealed a non-seminoma.

## DISCUSSION

We show for the first time that TM is more frequent in unaffected male relatives of TGCT cases than in healthy male controls. We also demonstrate that patients with a history of TGCT have a higher frequency of TM in their contralateral remaining testis than controls, supporting previous data that suggest an association between TGCT and TM. The higher frequency of TM in unaffected male relatives of TGCT cases than controls indicates that the association between TM and TGCT is not simply a consequence of the development or treatment of the testicular tumour. We have also demonstrated significant concordance of TM between relatives. The familial aggregation raises the hypothesis that TGCT and TM have a joint aetiology.

Our findings appear to be independent of the classification of TM. Many studies in the literature limit reports of TM to five or more microcalcifications (CTM). However, this cutoff is arbitrary and studies, which include LTM (one to four microcalcifications) also demonstrate an association with TGCT (Bennett *et al*, 2001; Middleton *et al*, 2002). For these reasons, we decided to count and analyse all TM (⩾1 microlith per testes) in this study. Therefore, the frequency of TM we report for healthy male participants is higher than in some previous studies, which report TM as ⩾5 microliths per testes (CTM) (Peterson *et al*, 2001; Serter *et al*, 2006). However, if we limit the definition of TM to ⩾5 microliths, the frequency of CTM is similar to other analyses of asymptomatic men at 6/101 (5.9%). Furthermore, if we apply this limit to all the data, the rate of CTM in TGCT cases is 32/169 (18.9%), for relatives is 11/58 (19.0%) and the differences between the cases and controls (18.7 *vs* 5.9%, age adjusted *P*=0.002) and relatives and controls (19 *vs* 5.9%, age adjusted *P*=0.01) remain statistically significant.

The familial clustering of TM demonstrated in this study could be due to shared environmental or shared genetic factors between relatives. Environmental factors, which may be involved in the genesis of TGCT are widely believed to occur *in utero* or in early childhood. We see concordance for TM in a variety of relative pairs, including father/son and son/father pairs and therefore prenatal environmental exposure is unlikely to account for the familial effect. Moreover, we do not show an excess of brother pairs in the TM concordant group, which we may have expected if the familiarity of TM was due to a shared environmental effect.

Overall, while not excluding an environmental component, these observations suggest that TM overlaps with TGCT susceptibility and that TM may be, at least in part, genetically determined. This is the first time such an observation has been made and suggests that TGCT susceptibility alleles may give rise to additional clinical phenotypes such as TM. However, in addition to the above, we would have expected that cases with a family history of TGCT would have a higher incidence of TM than cases without a family history. Similarly, we would have expected that relatives with familial TGCT would have a higher incidence of TM than those relatives with only one case in the family. While numerically this was the case in our study, the difference was not statistically significant. The small numbers of matched case–relative pairs in the analysis may have accounted for this and an additional larger series would be required to confirm this trend.

As with all studies that recruit volunteers as controls, there is a potential for bias. However, in this study, 56% of controls were nominated by previous research participants, usually a friend or unrelated relative (e.g. brother-in-law), and then approached to enter the study, thus limiting this effect. The remainder of the control group was derived from volunteers. As demonstrated, the overall rate of CTM among healthy controls in our study is similar to that reported previously (Peterson *et al*, 2001; Serter *et al*, 2006). Furthermore, there was no difference in the rate of TM reported between the two control sources (8/57 (14%) *vs* 10/44 (22.7%), *χ*^2^(1)=1.3, *P*=0.3). Interestingly, the frequency of CTM in our control population with a mean age of 42 years reflects that of the younger asymptomatic populations evaluated. This would suggest that frequency of TM does not change with age.

The study does not determine the temporal trends of TM. We examined the frequency of TM in the contralateral testis in patients with a previously diagnosed tumour; however, we did not have access to the original diagnostic ultrasound reports for comparison. Therefore, we cannot determine if the TM demonstrated in the contralateral testis was present at diagnosis.

Our study, similar to other studies that have investigated the association of TGCT with TM, does not show that TM is a pre-invasive lesion and additional longitudinal studies are required to determine if any of our participants with TM are subsequently diagnosed with TGCT.

In conclusion, we not only demonstrate that TM is more frequent in TGCT cases than controls, but importantly show that TM is more frequent in unaffected male relatives of TGCT cases. This would suggest that TM is a familial risk factor for TGCT and may have a joint aetiology. Furthermore, we demonstrate that TM aggregates in families, suggesting a common genetic susceptibility to TGCT. This may have implications for mapping and identification of TGCT genes.

## Figures and Tables

**Table 1:** Classification of TM

**Group**	**Number of microliths per testis**
None	0
Limited	1–4
Scant	5–24
Moderate	⩾25 but without areas of confluence
Too numerous to count	⩾25 and with areas of confluence

**Table 2:** Research participants, age at ultrasound, report of UDT and testis available for ultrasound

**Study group**	**Number of men examined by ultrasound**	**Mean age at ultrasound (range)**	**Surgery for undescended testis**	**Both testis examined**	**One testis examined**	**One testis examined with previous CIS or malignancy**	**Number of testes examined**
**TGCT Cases**	**169**	**47 (25–78)**	**21**	**7**	**158**	**4**	**176**
Subgroup A: TGCT-FH	41	47 (25–68)	3	1	39	1	42
Subgroup B: TGCT-S	128	47 (26–78)	18	6	119	3	134
							
**Relatives**	**58**	**44 (18–74)**	**2**	**57**	**1**	**0**	**115**
Subgroup C: Relative-FH	24	41 (18–74)	2	24	0	0	48
Subgroup D: Relative-S	34	46 (19–70)	0	33	1	0	67
							
**Control**	**101**	**42 (20–74)**	**2**	**101**	**0**	**0**	**202**
Total	328		25	165	159	4	493

FH=family history; S=sporadic.

**Table 3:** First tumour histology for index TGCT cases

	**TGCT-FH**			**TGCT-S**			**All cases**		
	**Number of cases**	**% known histology**	**Mean age at diagnosis**	**Number of cases**	**% known histology**	**Mean age at diagnosis**	**Number of cases**	**% known histology**	**mean age at diagnosis**
Seminoma	21	51.2	37	55	43.3	39	76	45.2	38
Non-seminoma	20	48.8	31	72	56.7	31	92	54.8	31
Unknown	0	—		1	—	20	1	—	20
									
Total	41	—	34	128	—	34	169	—	34

Percentage given for known histology.

**Table 4:** Testicular microlithiasis detected per study group

			**Unilateral TM**				**Bilateral TM^a^**				
**Study group**	**Subgroup**	**No TM**	**Limited**	**Scant**	**Moderate**	**TNTC**	**Limited**	**Scant**	**Moderate**	**TNTC**	**Total TM/total cases (% TM/group)**
Cases		107	30	15	9	7	0	1	0	0	62/169 (36.7)
	TGCT-FH	23	6	5	3	3	0	1^b^	0	0	18/41 (43.9)
	TGCT-S	84	24	10	6	4	0	0	0	0	44/128 (34.4)
											
Relatives		38	8	3	0	0	1	5	2	1	20/58 (34.5)
	Relative-FH	13	4	1	0	0	1	3	1	1	11/24 (45.8)
	Relative-S	25	4	2	0	0	0	2	1	0	9/34 (26.5)
											
Control		83	9	1	0	0	3	2	2	1	18/101 (17.8)

TNTC=to numerous to count.

a TM detected in both testes, highest degree of TM scored.

b The patient presented with metastatic seminoma and was treated with chemotherapy and radiotherapy alone. He had limited TM of the right testis and scant TM in the left. The testicular primary was never established in this patient.
